# Ang II Enhances Noradrenaline Release from Sympathetic Nerve Endings Thus Contributing to the Up-Regulation of Metalloprotease-2 in Aortic Dissection Patients' Aorta Wall

**DOI:** 10.1371/journal.pone.0076922

**Published:** 2013-10-23

**Authors:** Zhipeng Hu, Zhiwei Wang, Hongbing Wu, Zhimin Yang, Wanli Jiang, Luocheng Li, Xiaoping Hu

**Affiliations:** 1 Department of Cardiothoracic Surgery, Renmin Hospital of Wuhan University, Wuhan, Hubei Province, China; 2 Department of Cardiothoracic Surgery, Xiangyang Central Hospital, Xiangyang, Hubei Province, China; Max-Delbrück Center for Molecular Medicine (MDC), Germany

## Abstract

**Object:**

To test the hypothesis that angiotensin II (Ang II) could enhance noradrenaline (NA) release from sympathetic nerve endings of the aorta thus contributing to the up-regulation of matrix metalloproteinase 2 (MMP-2) during the formation of aortic dissection (AD).

**Methods:**

Ang II, NA, MMP-2, MMP-9 of the aorta sample obtained during operation from aortic dissection patients were detected by High Performance Liquid Chromatography and ELISA and compared with controls. Isotope labelling method was used to test the impact of exogenous Ang II and noradrenaline on the NA release and MMP-2, MMP-9 expression on Sprague Dawley (SD) rat aorta rings in vitro. Two kidneys, one clip, models were replicated for further check of that impact in SD rats in vivo.

**Results:**

The concentration of Ang II, MMP-2, 9 was increased and NA concentration was decreased in aorta samples from AD patients. Exogenous Ang II enhanced while exogenous NA restrained NA release from aortic sympathetic endings. The Ang II stimulated NA release and the following MMP-2 up-regulation could be weakened by Losartan and chemical sympathectomy. Beta blocker did not influence NA release but down-regulated MMP-2. Long term in vivo experiments confirmed that Ang II could enhance NA release and up-regulate MMP-2.

**Conclusions:**

AD is initiated by MMP-2 overexpression as a result of increased NA release from sympathetic nervous endings in response to Ang II. This indicates an interaction of RAS and SAS during the formation of AD.

## Introduction

Both renin–angiotensin system (RAS)and sympathetic adrenergic system(SAS)participate in the pathological process of aortic dissection (AD). By now, the two most widely used AD animal models are based on subcutaneous injection of angiotensin II (Ang II) combined with apolipoprotein-E (ApoE) knock-out or beta-aminopropionitrile monofumarate (BAPN) pretreatment [1], [2]. Habashi JP [3] reported that Losartan, an AT1 antagonist, could prevent major life-threatening manifestation such as AD in a mouse model of Marfan syndrome. Clinical trials also support their finding [4], [5]. On the other hand, adrenergic antagonists such as β-blockers were also widely used for preventing AD and have shown the nice effect [6], [7]. Their rationale includes reduction in arterial pressure and heart rate leading to decreased shear stress on the aorta and slower aortic root growth [8].

Recently published studies are somewhat not accordant to the above conclusion. Both Ang II and noradrenaline (NA, the adrenergic transmitter) were thought to have the potential of inducing AD by increasing blood pressure in ApoE deficient or BAPN pretreated animals. But it can be debate that only the blood pressure elevation induced by Ang II can induce model AD [1], [9], [10]. So whether RAS and SAS do affect AD formation needs further research.

Matrix metalloproteinase 2 (MMP-2) and Matrix metalloproteinase 9 (MMP-9) are important metalloproteinases (MMPs) which can degrade extracellular matrix (ECM) and are differently regulated by RAS and SAS [11]. The different result of Ang II and NA in inducing model AD can be attributed to their differences in regulating MMPs.

It is widely accepted that the Ang II blood concentration is elevated in AD patients and in several animal models. We recently observed an increase of Ang II and a decrease of NA in the AD patients' aorta. A general agreement exists that Ang II can enhance NA release. This effect is demonstrated by several observations derived from experiments in different tissues like peripheral vascular, prostate and preoptic recess [12], [13], [14], [15], [16]. We hypothesized that Ang II could also enhance NA release in the aorta. Furthermore, we hypothesized that an interaction between RAS and SAS might exist in regulating MMPs,and designed experiments to check them.

## Methods

### Human aorta samples

The study protocol was approved by the Medical Ethics Committee of the Renmin Hospital of Wuhan University, and informed written consent was obtained from each subject. 16 thoracic aortic dissection (TAD) patients without phenotypic characteristics of any of the known genetic disorders, such as Marfan's syndrome, Loeys-Dietz syndrome, Turner's syndrome or such family anamnesis, were included in the experimental group. Of them, 10 were male, 8 were combined with hypertension, and average age was 46±6.3 years. All of them were made a definite diagnosis by CT and operation. Full thickness aortic wall specimens were harvested in operation and stored in −80°C fridge. Abdominal aortic wall specimens from 3 kidney donors and 6 thoracic aortic wall specimens from aortic valve replacement patients were obtained in the control group. Of them, 6 were male, 4 were combined with hypertension, and average age was 49±11.6 years. None of the people in the control group were diagnosed for the following diseases or experienced such disease history: Marfan's syndrome, Loeys-Dietz syndrome, Turner's syndrome or such family anamnesis, aorto-arteritis, suffering with other known heart, lung, kidney or liver disease.

### Short term *in vitro* experiments

#### Animals and aorta tissue preparation

The study protocol (including the in vitro and the in vivo experiments) was approved by the Ethical Committee of the Renmin Hospital of Wuhan University, and all animal handling was performed in accordance with the Wuhan Directive for Animal Research and the current Guidelines for the Care and Use of Laboratory Animals published by the National Institutes of Health (NIH publication no. 85–23, revised 1996). Rats were killed by decapitation after successfully anesthetized with 1% phenobarbital after two weeks pretreatment. Unnecessary suffering was avoided as far as possible.

The experiments were performed on 36 8-week-old Sprague-Dawley male outbred rats. All animals were divided into 5 groups: A. group without pretreatment (n = 12); B. Losartan pretreatment group (40 mg/kg/day) (n = 6); C. Chemical sympathectomy group (n = 6) (Guanethidine, Sigma Chemical Co, 50 ug/g/d subcutaneously injected, 5 days per week); D. Doxazosin pretreatment group (1 mg/kg/d) (n = 6); E. Metoprolol pretreatment group (100 mg/kg/day) (n = 6).

After animals were anesthetized (1% phenobarbital, 30 mg/kg) and killed, an incision was made on the left side of the chest and the ascending aorta was dissected free. The isolated aorta was transferred to a Petri dish containing pre-warmed physiological salt solution (PSS). Excess fatty tissue was removed and the aorta was cut into 2 mm thick aorta rings. Aorta rings were collected for further experiments from each rat and were immediately placed in 0.1M Perchloric acid (HClO_4_) solution and kept overnight.

#### Noradrenaline radiolabeling and measurement of the radioactivity

The methods for noradrenaline radiolabeling and measurement of the radioactivity were made as described [14], [17]. Briefly, the aortic rings were immersed in Krebs physiologic salt solution, and then were incubated with 3H-noradrenaline (3H-NA, specific activity30–50 Ci/mmol, Amersham Pharmacia Biotech Pty Ltd, UK) for 1 h in small beakers containing 5 ml medium with (−) -3H-NA (0.23 umol/l). Aorta rings were then washed for 90 min before experimental procedures. After the washout period, the intrinsic sympathetic nerves of the aorta ring preparations were subjected to two 60 s periods of electrical field stimulation (1 ms pulses, 5 Hz, 20 V, 400 v/m) [14]. The stimulation was given at 30 min after the washout period. The effects of Ang II and NA on the resting and stimulation-induced effluxes of radioactivity were examined by adding the drugs (Ang II: 0.1 µmol/l;and NA, 1 mmol/l; decided by preliminary experiment with dose-response curve and the concentration which induced maximal effect in the dose-response curve was used in the following experiments) to the PSS superfusate of the aortic rings. The drugs then remained present for the duration of the experiment. The superfusate from the aorta ring preparations was collected at 3 min intervals by an automated ISCO Retriever IV fraction collector (ISCO Inc.). Each 3 min (6 ml) fraction of superfusate was mixed with 4 ml of scintillant (Ultima Gold, Packard Bioscience BV, Groningen, The Netherlands) and the radioactivity present determined by liquid scintillation counting. External automatic standardization was utilized to correct for counting efficiency and the data were expressed in disintegrations per minute (d.p.m.). Then the vascular rings were heated (100°C in a dry bath) with 200 µl H2O2 (30 Vol) plus 200 µl HClO4, 30% during 15 min. Scintillation fluid was added into the solubilized samples, and the radioactivity was measured in a Packard 2000 spectrometer. After correction by vessel weight, the percentage of 3H-NA release in a certain period of time was calculated according to the expression:
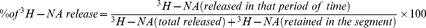



### Long term *in vivo* experiments

#### Animals preparation and grouping

The experiments were performed on 36 8-week-old Sprague-Dawley male outbred rats. All animals were divided into 3 groups: A. Control group (did not accept any additional treatment except for the same feeding and housing as the other groups) (n = 18); B. 2K1C group (Two kidney, one clip; the renal artery stenosis surgery was done to elevate the endogenous Ang II according to the method described by Head GA et al [18]) (n = 18); C. 2K1C+Chemical sympathectomy group (n = 18) (Guanethidine, Sigma Chemical Co, 50 ug/g subcutaneously injected, 5 days per week since one week after the surgery).

#### Samples harvesting

On the last day of the 4th, 7th and 10th week, 6 rats from each group were killed in anesthesia (1% phenobarbital, 30 mg/kg) and then the thoracic aorta was harvested for detecting Ang II, NA and MMPs.

### Additional methods

#### Ang II and NA concentration in the aorta samples

Small segments of the aorta were immediately placed in 0.1M Perchloric acid (HClO4) solution after harvest and kept overnight. Aliquots of the extract were stored at −80°C until analysis. Ang II and NA concentration was assayed by reverse-phase high performance liquid chromatography (HPLC) and HPLC with electrochemical detection.

#### MMP-2 and MMP-9 detection

Samples prepared for ELISA were treated according to the means used by Møller MN et al [19]. MMP-2 and MMP-9 in the aorta samples were determined by ELISA. The analyses were performed according to the manufacturer's instructions. The analyses were performed in duplicate and the mean value used for the subsequent calculations.

### Statistical Analysis

Standard statistical methods were used for the calculation of means and standard deviations. The data was analyzed by an unpaired Student's t-test to determine the difference between groups and analysis of variance (ANOVA) followed by Tukey Test was applied when unpaired t test is unfit to be used. All statistical analysis was performed with Graphpad Prism software (5^th^ version). *P* value less than 0.05 was regarded as significant.

## Results

### Elevated concentration of Ang II, MMP-2, 9 and decreased NA concentration in aorta samples from AD patients

16 AD patients were included in this study. Baseline data were given in table 1.

**Table 1:** Baseline data of studyied population.

	AD (n = 16)	Control (n = 9)	*P* Value
Male (n)	10	6	*P* = 0.835
Age (years)	46±6.3	49±11.6	*P* = 0.4071
Hypertension (n)	8	4	*P* = 0.7896

**Figure 1:**
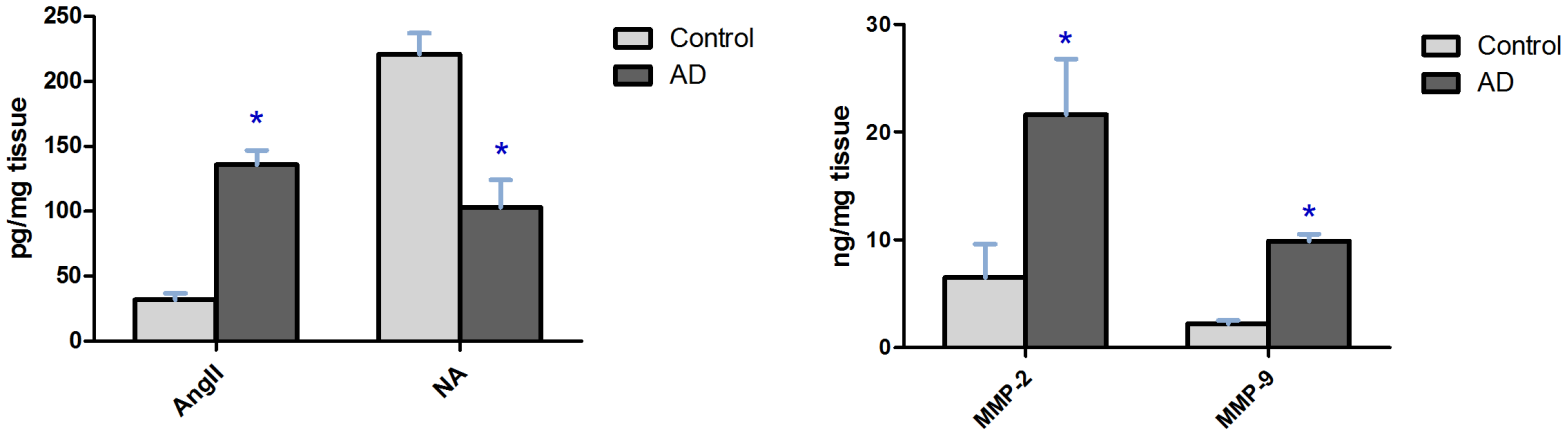
Tissue concentration of AngII, NA and MMP-2, 9 in human aortic tissue samples. Data shown are means ±SD. *P<0.01 related to control.

High Performance Liquid Chromatography was used for detecting tissue Ang II and NA concentration in aorta samples. As it was represented in Figure 1, Ang II concentration was higher (136±11 VS 32±5, P<0.01) and NA concentration was lower (103±21 VS 221±16, P<0.01) in the AD patients than in the control group. Higher MMP-2 (21.6±5.2 VS 6.5±3.1, P<0.01) and MMP-9 (9.9±0.6 VS 2.2±0.3, P<0.01) expressions were detected by Elisa in the AD patients aorta wall than in controls.

### Impact of Ang II and NA on NA release *in vitro*

**Figure 2:**
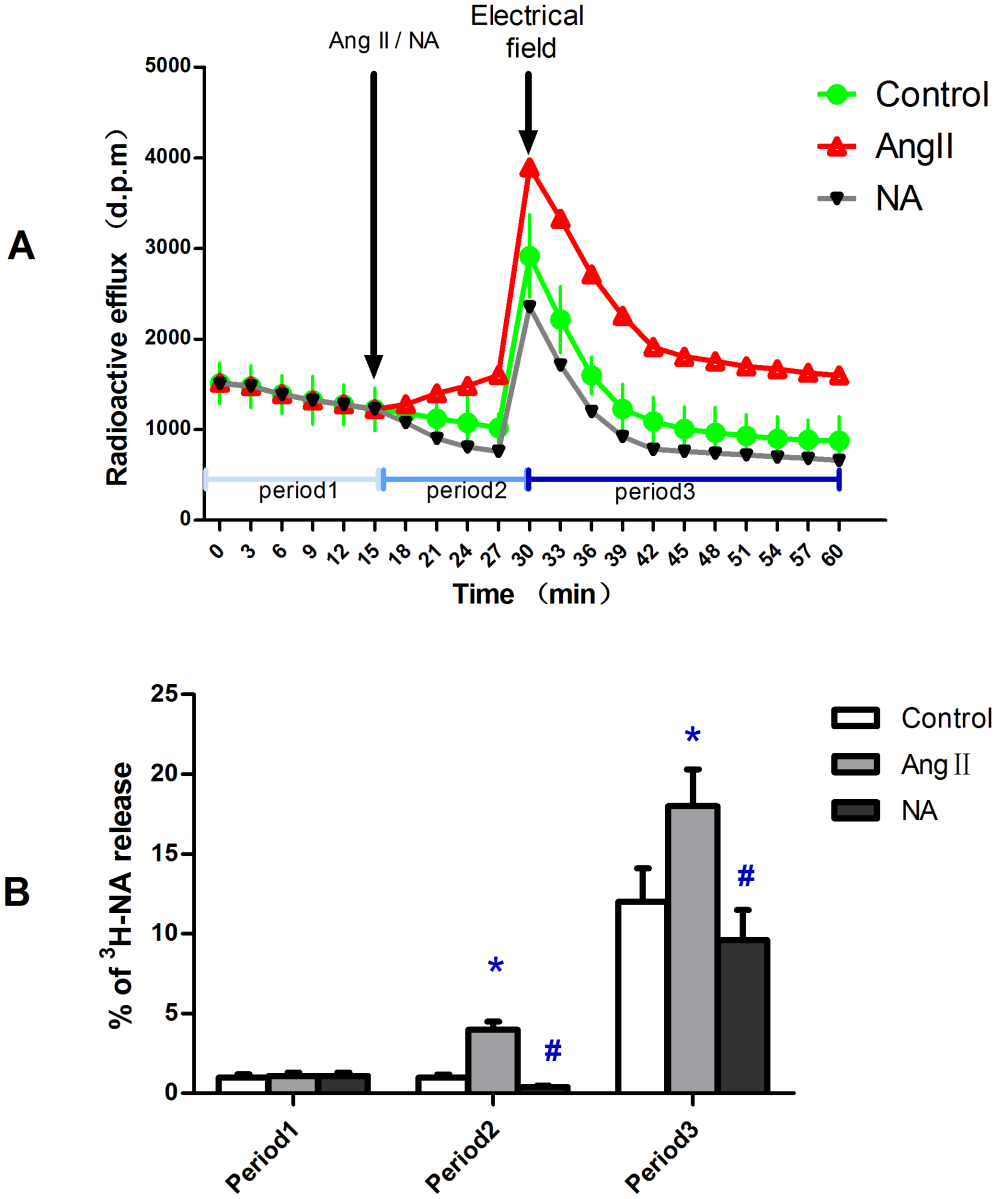
In vitro experiments on SD rat aorta rings investigating NA release using isotope labelling. Ang II or NA was added 15 min before electrical field stimulation. In each period of time the release fraction was calculated according to the expression given formerly. A: release curve graph of ^3^H-NA. B: statistical chart of % ^3^H-NA release in three periods of time. Period1: spontaneous release; period2: spontaneous release after Ang II or NA were added in; period3: electrical field stimulated release after Ang II or NA were added in. Data shown are means ±SD. *P<0.01 related to control, ^#^P<0.05 related to control.

There was no difference in spontaneous NA release among three groups. The spontaneous NA release (period1 in figure 2A) was enhanced after Ang II was added in but was slightly weakened after NA was added in (period2 in figure 2A). The same trend was also observed in the electrical stimulated release period (period3 in figure 2A). This indicates an enhancement of Ang II and a feedback effect of NA on NA release in the aorta. (Figure 2)

### The impact of chemical sympathectomy and receptor antagonisms on the Ang II stimulated NA release and MMP-2, MMP-9 expression *in vitro*

**Figure 3:**
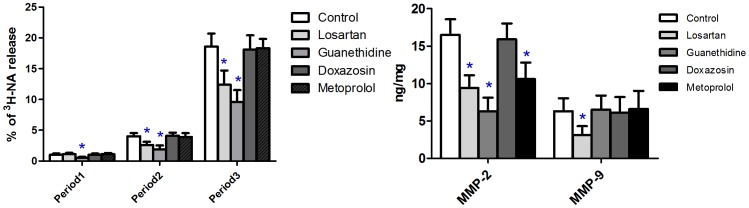
In vitro experiments on SD rat aorta rings investigating AngIIstimulated NA release and MMP-2, 9 expression after chemical sympathectomy or receptor antagonism. Data shown are means ±SD. *P<0.01 related to control.

To further investigate the enhancement effect of Ang II on NA release, aorta rings from the control group, chemical sympathectomy group and receptor antagonisms pretreated groups were also radiolabeled and detected for ^3^H-NA release. The percentage of ^3^H-NA release in each period was calculated using the former given expression and compared respectively. In the chemical sympathectomy group, NA release was restrained in all three periods(period1: 0.5±0.16% VS 1.0±0.2%; period2: 1.9±0.6% VS 4.0±0.5%; period3: 9.6±1.9% VS 18.6±2.1%; *P*<0.01 in each period of time), followed by a down-regulation of MMP-2 (6.3±1.8 VS 16.5±2.1, *P*<0.01). Losartan weakened the Ang II stimulated NA release in both period2 (2.6±0.5% VS 4.0±0.5%, *P*<0.01) and period3 (12.4±2.3% VS 18.6±2.1%, *P*<0.01). Both MMP-2 (9.4±1.7 VS 16.5±2.1, *P*<0.01) and MMP-9 (3.1±1.2 VS 6.3±1.7, *P*<0.01) were down regulated in the losartan pretreated group. Both doxazosin and metoprolol did not show any restrain effect on the Ang II stimulated NA release in any period of time (Doxazosin: period1: 1.0±0.2% VS 1.0±0.2%; period2: 4.1±0.5% VS 4.0±0.5%; period3: 18.1±2.3% VS 18.6±2.1%; *P*>0.05 in each period of time. Metoprolol showed the same trend). But the later one showed a restrain effect on the expression of MMP-2 (10.6±2.2 VS 16.5±2.1, P<0.01). (Figure 3)

### Impact of Ang II elevation on the NA release and MMP-2, MMP-9 expression in vivo

**Figure 4:**
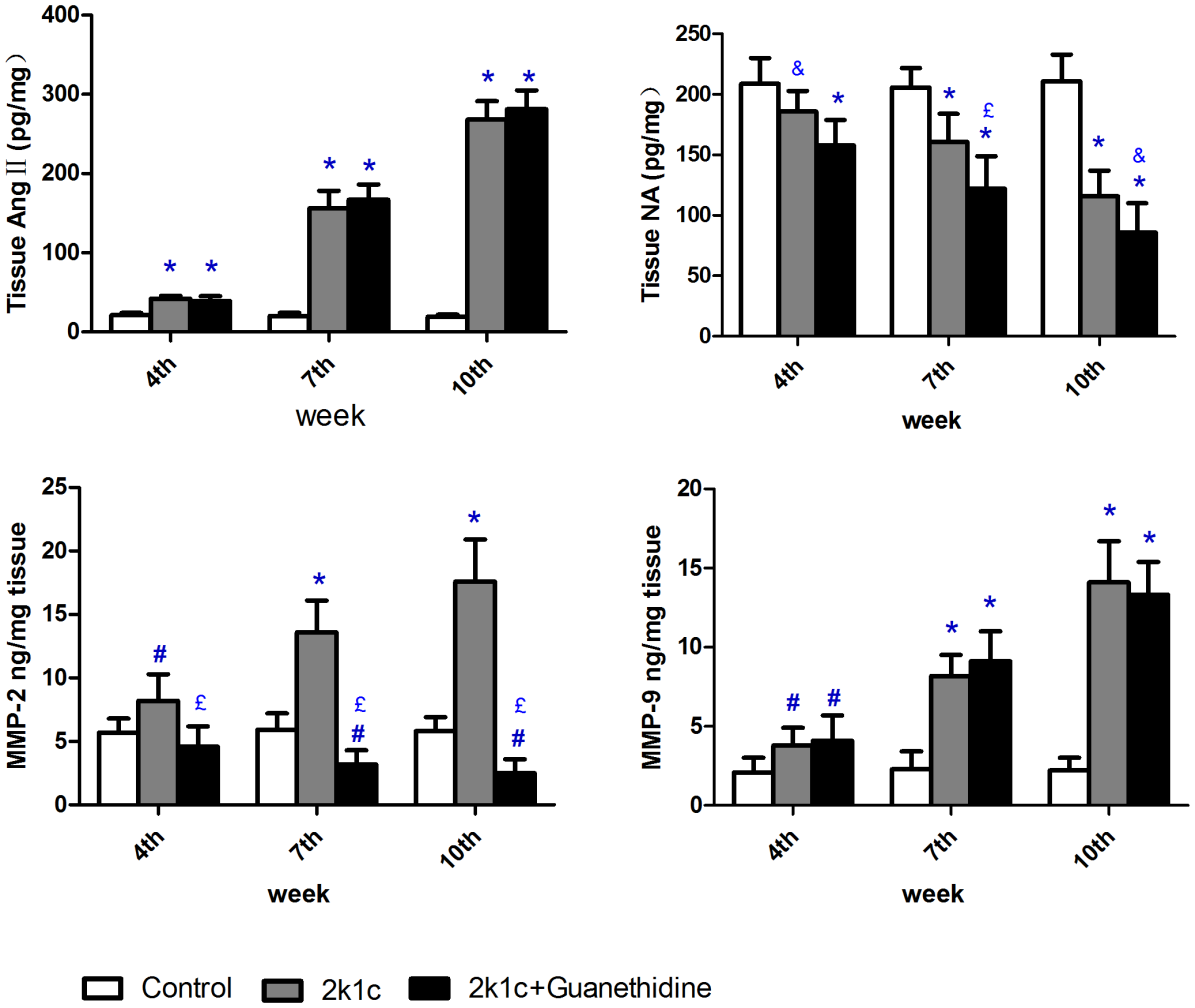
Impact of AngII elevation with or without chemical sympthectomy on NA release and MMP-2, 9 expression in SD rats in vivo. Data shown are means ±SD. *P<0.01 related to control, ^#^P<0.05 related to control, ^£^<0.01 related to 2k1c, ^&^P<0.05 related to 2k1c.

In the long term in vivo experiment, Ang II elevation was successfully induced by 2k1c. Following the gradual elevation of Ang II (42±3.6, 156±22.0, 268±23.0 at the end of the 4^th^, 7^th^, 10^th^ week, respectively; all values in pg/mg), the tissue NA decreased gradually (186±17, 161±23.0, 116±21.0 at the end of the 4^th^, 7^th^, 10^th^ week, respectively; all values in pg/mg). Chemical sympathectomy had no significant further influence add to 2k1c on the Ang II tissue concentration all the time (2k1c+chemical sympathectomy VS 2k1c, pg/mg: 4^th^ week:39±6 VS 42±3.6; 7^th^ week:167±19 VS 156±22; 10^th^ week:281±24 VS 268±23; *P*>0.05 on each time point). On the other hand, Chemical sympathectomy significantly strengthened the reduction effect of 2k1c on tissue NA concentration (2k1c+chemical sympathectomy VS 2k1c, pg/mg: 4^th^ week:158±21 VS 186±17, *P*<0.05; 7^th^ week:122±27 VS 161±22, *P*<0.01; 10^th^ week:86±24 VS 116±23, *P*<0.05). 2k1c procedure showed a significant enhancing effect on both MMP-2 (2k1c VS control, ng/mg: 4^th^ week:8.2±2.1 VS 5.7±1.1; 7^th^ week:13.6±2.5 VS 5.9±1.3; 10^th^ week:17.6±3.3 VS 5.8±1.1; *P*<0.01 on each time point) and MMP-9 (showed the same trend as MMP-2) on each time point. When chemical sympathectomy was added to 2k1c, MMP-2 expression was restrained significantly (4.6±1.6, 3.2±1.1 and 2.5±1.1 at the end of the 4^th^, 7^th^, 10^th^ week, respectively; ng/mg; *P*<0.01 compared with 2k1c group on each time point) but MMP-9 expression was still up-regulated(4.1±1.6, 9.1±1.9 and 13.3±2.1 at the end of the 4^th^, 7^th^, 10^th^ week, respectively; ng/mg; *P*>0.05 compared with 2k1c group on each time point). (Figure 4)

## Discussion

The key finding derived from the present work is that the elevated circulating Ang II enhances NA release from sympathetic nerve endings thus up-regulating MMP-2, which is known to degrade the extracellular matrix, including collagen and elastin, and contributes to AD progression.

That Ang II enhances NA release has been proven in several organs but not yet in the aortic wall in connection with aneurysm development [12], [13], [14], [15], [16], [20]. The interaction between RAS and SAS is involved in many pathological processes [21], [22], [23]. Both RAS and SAS are important in maintaining physiological state of the aorta. It is important to reveal whether the interaction between RAS and SAS participates in the initiation and progression of AD. Isotope labelling is a useful method for studying NA release in a certain tissue and has been used in many studies [14], [17], [24], [25], [26]. We can investigate not only the spontaneous NA release but also the stimulated NA release. Electrical field is a commonly used approach to stimulate isotope labeled substance release. This approach can accelerate ^3^H-NA release and reach a more significant difference of release percentage in a limited period of time. A proper stimulating intensity is important and our experiment took examples from Fabiani ME's study [14] to determine the intensity. By means of isotope labelling, we proved such an interaction does exist in the aorta and confirmed that conclusion by receptor antagonism and chemical sympathectomy. Losartan, an ATR1 antagonist, was observed to block the effect of Ang II, as it was suggested by former studies [27], [28]. Guanethidine destroys cell bodies of sympathetic ganglia and produces permanent terminal axon degeneration that reached the blood vessels thus conduct a chemical sympathectomy [29]. This feature enables us to further check the potential of Ang II in stimulating NA release in our experiment, both in vitro and in vivo.

Fragmentation of the elastic fibers and aortic dilation are associated with the local elevation of metalloprotease (MMPs) expression and activity. MMPs leads to degeneration of the ECM and destruction of the vascular framework. MMP-2 and MMP-9 are the main kinds of MMPs which have been implicated in AD formation [30], [31], [32]. Both MMP-2 and MMP-9 are also overexpressed in different Ang II infusion induced aortic aneurysm and aortic dissection models [33]. This kind of overexpression is attenuated in β-Arrestin-2 (βarr2) deficiency mice by the interruption of Ang II–AT1a-βarr2- COX2 pathway [34]. Only MMP-9 overexpression is attenuated in AKT2 deficiency mice because AKT2 regulates FOXO1 which bind to the promoters of MMP-9 [35]. MMP-9 was mainly derived from neutrophils and was regulated by Ang II via affecting neutrophils filtration [1]. But how RAS and/or SAS influence MMP-2 during the formation of AD has not been revealed. Our study observed that NA release could up-regulate MMP-2 and this could be weakened by beta1 receptor antagonism, in accordance with former studies [36], [37]. It was reported formerly that circulating noradrenaline has a feedback effect on sympathetic outflow [38]. Our study also observed a feedback effect of circulating noradrenaline on the NA release from sympathetic nerve endings. Furthermore, such feedback effect can also affect MMP-2 expression. That Ang II stimulates NA release and MMP-2 expression indicates an interaction between RAS and SAS on the regulation of MMP-2. Our finding is agreed with Dab H et al [39] and gives further explanation. So our findings also provide a probable explanation for the different result of Ang II and NA in inducing AD model.

For inducing circulating Ang II elevation, there are at least three models, including Ang II infusion model, 2k1c model and aortic coarctation model. Each model has its advantages and disadvantages. Ang II infusion ensures better uniformity in models but prevents us from extending experiment time and increasing time points for comparison. Both 2k1c and aortic coarctation can provide a long period of Ang II elevation but 2k1c is more likely to induce a higher Ang II level [18], [20], [40]. So in our experiment, 2k1c model was selected and an elevation of tissue Ang II concentration has been successfully induced following the elevated circulation Ang II.

A limitation of our study is the absence of observing tissue fluid NA concentration in real time and dynamically by a microdialysis system [23]. It further proves that this change of NA release affects MMP-2 expression. Another limitation is the absence of detection for MMP-2 and MMP-9 activity, which is also very important in the AD initiation and progression [41].

In conclusion, AD is initiated by MMP-2 overexpression as a result of increased NA release from sympathetic nervous endings in responding to Ang II. This indicates an interaction of RAS and SAS during the formation of AD.
